# Case report: Treatment of metastatic dedifferentiated chondrosarcoma with pembrolizumab yields sustained complete response

**DOI:** 10.3389/fonc.2022.991724

**Published:** 2022-11-17

**Authors:** Amisha Singh, Steven W. Thorpe, Morgan Darrow, Janai R. Carr-Ascher

**Affiliations:** ^1^ Department of Internal Medicine, University of California, Davis Medical Center, Sacramento, CA, United States; ^2^ Department of Orthopaedic Surgery, University of California, Davis Comprehensive Cancer Center, Sacramento, CA, United States; ^3^ Department of Pathology and Laboratory Medicine, University of California, Davis Medical Center, Sacramento, CA, United States; ^4^ Division of Hematology and Oncology, University of California, Davis Comprehensive Cancer Center, Sacramento, CA, United States

**Keywords:** case report, pembrolizumab, immunotherapy outcomes, dedifferentiated chondrosarcoma (DDCS), chondrosarcoma

## Abstract

Dedifferentiated chondrosarcomas (DDCS) are aggressive tumors with poor outcomes. Treatment of localized DDCS is primarily surgical, though most patients present with unresectable or metastatic disease. Systemic treatment options for advanced DDCS are limited, and the benefits of chemotherapy in this patient population remain controversial. Among other systemic therapy options, there is emerging clinical evidence to support the use of immunotherapy in patients with advanced DDCS. However, studies regarding the efficacy of immunotherapy in advanced DDCS are limited. Here, we present the case of a patient with metastatic, programmed death-ligand 1 (PD-L1)-positive DDCS treated with pembrolizumab who showed a sustained complete response for 24 months after initiation of therapy. To our knowledge, this case represents one of few documented cases of metastatic chondrosarcoma with sustained response to immunotherapy. The impressive response seen with PD-L1 inhibition in our patient indicates that immunotherapy is a successful treatment option in a subset of DDCS patients, and further investigation is needed to identify potential responders to immunotherapy.

## Introduction

Dedifferentiated chondrosarcomas (DDCS) are rare, aggressive mesenchymal tumors that account for up to 11% of all chondrosarcomas and 1%–2% of all primary bone tumors (1, 2). These tumors are characterized by well-differentiated, low-grade cartilaginous components lying adjacent to high-grade, dedifferentiated mesenchymal cells (3). Patients commonly present in the fifth decade of life with a rapidly growing soft tissue mass in the femur, pelvis, or humerus and imaging classically reveals a large unmineralized soft tissue mass with cortical infiltration and osteolytic bone destruction (4). The prognosis for DDCS is dismal, with a median overall survival (mOS) of 5–13 months and 5-year survival rates ranging from 5% to 25% (1, 5–7). Studied prognostic factors associated with worse survival include larger tumors, unresectable disease, and presence of metastases at presentation (5, 8).

Currently, wide surgical resection remains the mainstay of treatment for localized DDCS. Unfortunately, many patients present with unresectable or metastatic disease not amenable to surgical removal (6, 9) or rapidly develop progression of disease. Systemic treatment options for these patients are limited. Furthermore, as these tumors are composed of a dense extracellular matrix, DDCS are poorly vascularized and resistant to chemotherapy, compounding their poor prognosis (6, 7, 10, 11). Given the limited benefit of chemotherapy demonstrated in DDCS, and with the growing success of immunotherapy in treatment of other aggressive malignancies, there is increasing interest in identifying patients who may respond to immunotherapy.

Here, we present the case of a patient with a metastatic, programmed death-ligand 1 (PD-L1)-positive, dedifferentiated chondrosarcoma treated with palliative resection followed by pembrolizumab. The patient demonstrated a remarkable response with regression of metastatic foci and a sustained complete response for 24 months. To our knowledge, this case represents one of few documented cases of metastatic DDCS with sustained response to immunotherapy (12).

## Case description

**Figure 1:**
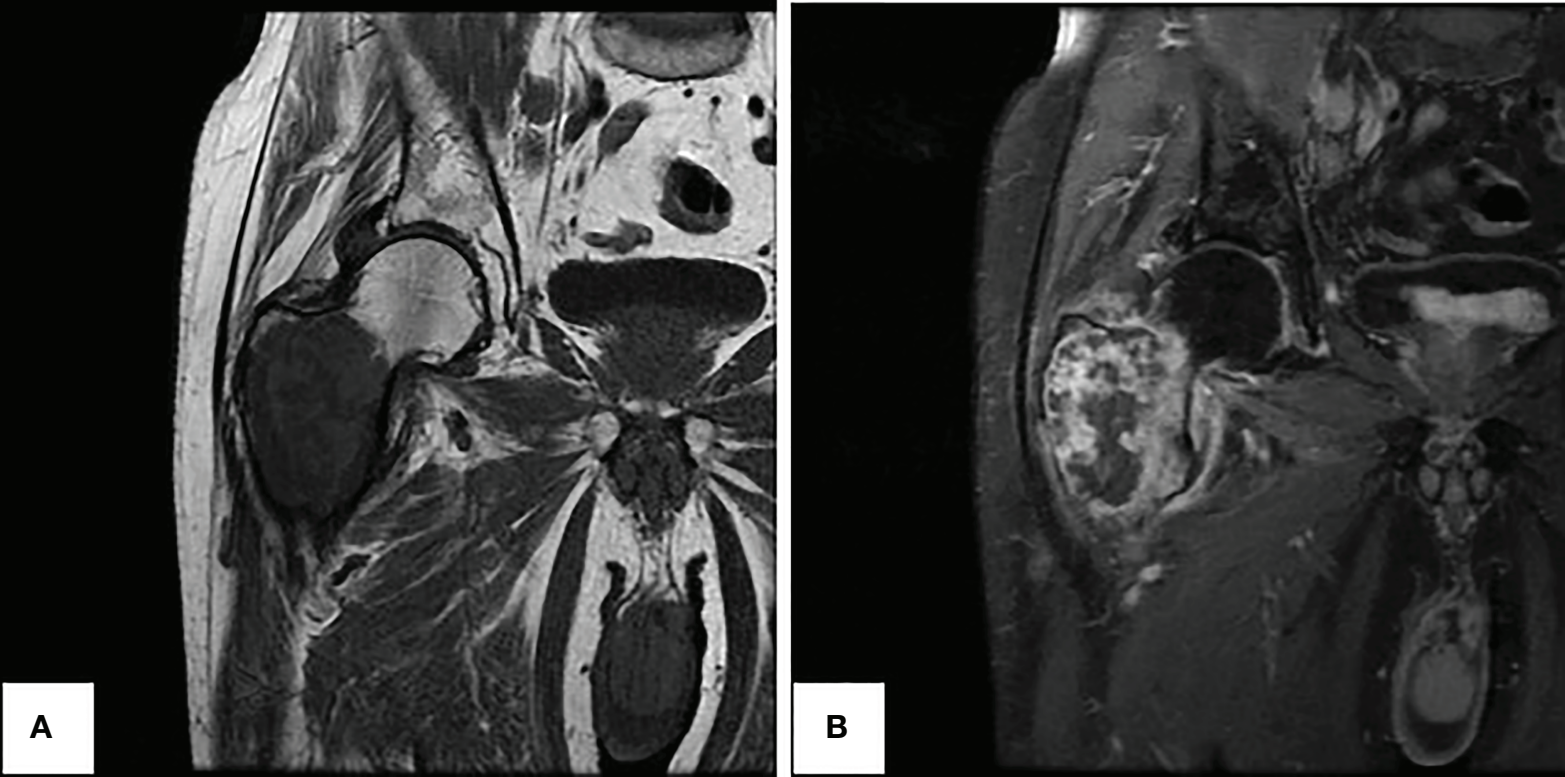
Preoperative MRI of right femur demonstrating large femoral ltyic mass. **(A)** Coronal view in T1 sequence (left). **(B)** Coronal view in T1 FAT-SAT sequence with algorithm (right).

**Figure 2:**
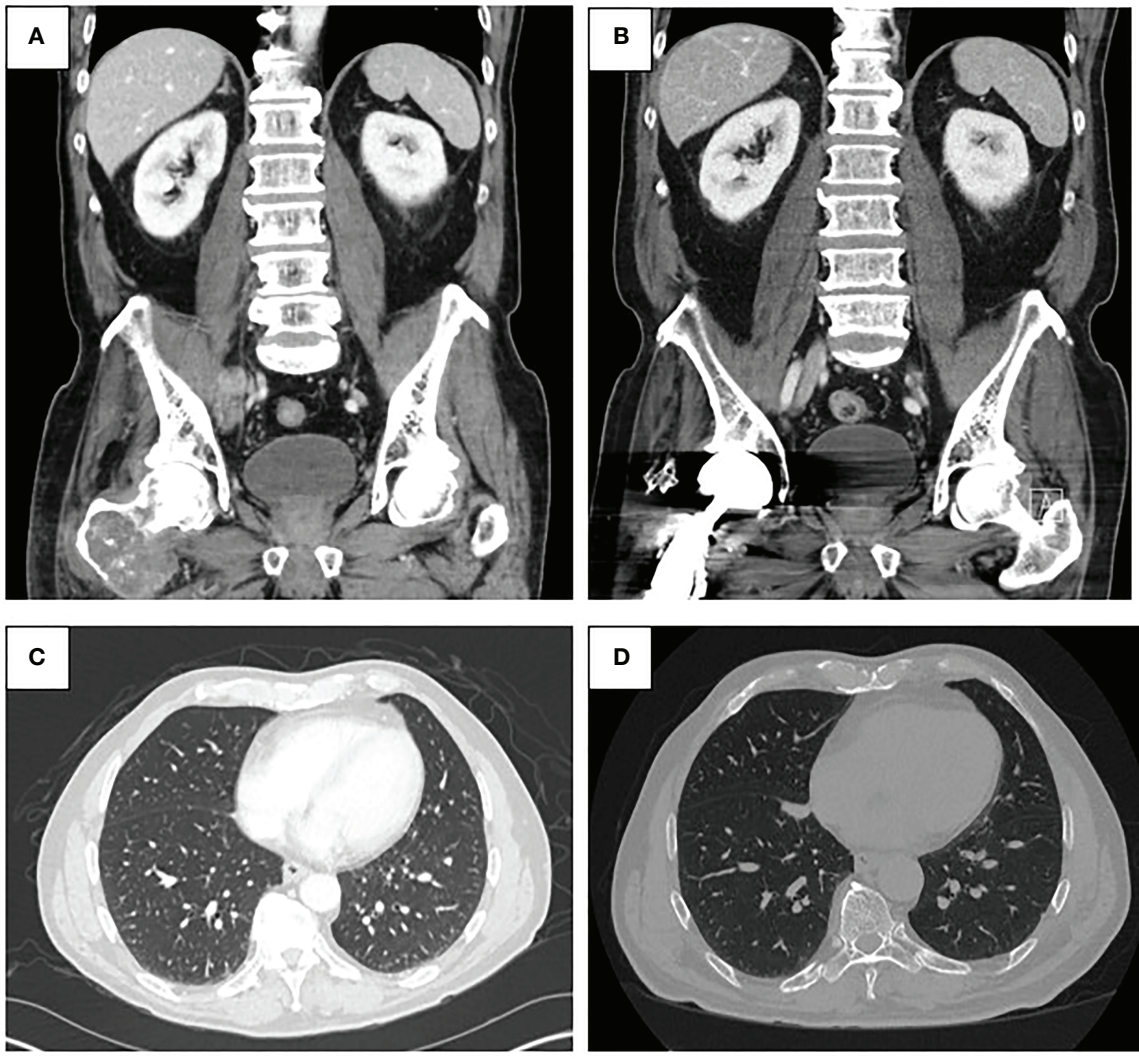
**(A)** Pre-treatment CT abdomen and pelvis demonstrating malignant pelvic lymph node involvement (top left). **(B)** Post-treatment CT abdomen and pelvis from January 2022 demonstrating resolution of pelvic lymphadenopathy (top right). **(C)** Pre-treatment CT chest demonstrating lung metastases (bottom left). **(D)** Post-treatment CT chest from January 2022 demonstrating regressed lung nodules (bottom right).

A 70-year-old man presented to his primary care physician for neck and back pain following a mechanical ground level fall. The patient had a past medical history significant for hypertension, hyperlipidemia, and Type 2 diabetes (hemoglobin A1c 6.9%). He endorses a family history of coronary artery disease and his maternal grandmother had breast cancer at an advanced age. He had no current or prior smoking history. He was found to have a right proximal femoral lytic lesion on x-ray and, therefore, was evaluated by an orthopedic oncologist. The physical exam was limited by pain and non-weight-bearing status; therefore, gait was not assessed. The distal neurovascular exam was normal with 5/5 strength in the tibialis anterior, extensor hallucis longus, and gastrocsoleus complex. He had an intact sensory exam in the sural, saphenous, superficial peroneal, deep peroneal, and tibial nerve distributions. 2+ posterior tibial and dorsalis pedis pulses were palpated. An MRI demonstrated a large mass measuring 9.7 × 6.7 × 4.5 cm, centered in the right proximal femur with a large extracortical soft tissue component extending posteriorly that measured 4.3 × 3.4 cm (**Figures 1A, B**). The patient underwent ultrasound-guided biopsy of the femoral mass with pathology revealing a high-grade sarcoma. MRI of the right lower extremity also revealed a concerning 2.1-cm right external iliac lymph node. Staging workup with CT chest/abdomen/pelvis showed multiple lung nodules and a 4.3 × 2.3 cm right pelvic sidewall soft tissue mass suggestive of metastatic disease (**Figures 2A, C**). Bone scan showed activity in the right proximal femur, but no other areas.

## Diagnostic assessment and treatment

**Figure 3:**
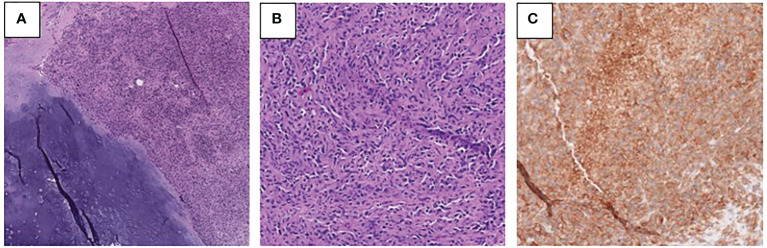
**(A, B)** Photomicrographs of the right femur tumor showing low-grade chondrosarcoma with adjacent dedifferentiated component **(A)** and the high-grade dedifferentiated component **(B)** (H&E). **(C)** PD-L1 immunohistochemical stain performed on the right femur tumor demonstrates membranous immunoreactivity in tumor cells.

The patient subsequently underwent palliative resection of the femur mass, right proximal femur replacement, and hemiarthroplasty of the right hip due to severe pain and risk of fracture. Pathology of the resected mass showed juxtaposition of an undifferentiated pleomorphic sarcoma with a low-grade chondrosarcoma, consistent with the diagnosis of dedifferentiated chondrosarcoma (**Figure 3**). Necrosis was present in 30% of the sample. Focal involvement of the superior soft tissue margin was noted. Next generation sequencing from Tempus testing of the mass showed microsatellite stability, p53 p.W91 loss-of-function mutation, NRAS p.G13R missense variant, TERT c.-124C>T variant, and a promoter mutation. PD-L1 positivity was measured to be 95% in the tumor membrane and 20% in tumor-associated immune cells. An IDH1 mutation was not identified.

With a diagnosis of metastatic dedifferentiated chondrosarcoma, our patient had a poor prognosis with limited treatment options. Previous data regarding treatment of DDCS are limited but demonstrate poor response rates and resistance to chemotherapy, as seen with other chondrosarcomas (6, 10–14). With the understanding that chemotherapy has limited efficacy in chondrosarcomas and is of questionable benefit in dedifferentiated chondrosarcoma, the patient opted for immunotherapy after considering the typical side effect profiles of chemotherapy versus immunotherapy.

In August 2020, the patient started treatment with intravenous pembrolizumab 200 mg every 3 weeks. Repeat imaging with a CT chest/abdomen/pelvis was obtained 5 weeks after initiation of therapy and showed a stable necrotic right pelvic lymph node and resolution of multiple lung nodules; one lung nodule previously noted to be 5 mm was noted to be 4 mm in size (**Figures 2B, D**). Given the positive response, the patient was continued on pembrolizumab treatment. In June 2021, a repeat CT scan demonstrated no metastatic disease in the abdomen or pelvis and showed an unchanged 4-mm pulmonary nodule.

The intention was to treat the patient with a total of 2 years of pembrolizumab, but this was discontinued after 29 cycles for steroid refractory, grade 3 immunotherapy-induced psoriatic arthritis that briefly limited his ability to work. The course was also complicated by Grade 1, asymptomatic hypothyroidism, treated with levothyroxine. Throughout the treatment, the patient developed hyperglycemia in the context of long-standing type 2 diabetes. This was unlikely related to immunotherapy and did not require insulin.

After recovery from surgery, he returned to his daily activities. He is a small business owner and has returned to working daily, exercising, and traveling. Despite discontinuing pembrolizumab, he has continued to have a complete response on surveillance imaging.

**Figure 4:**
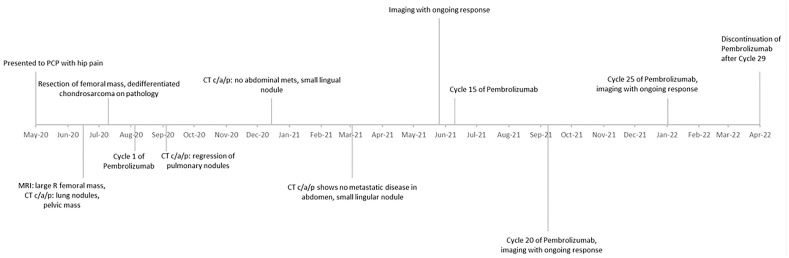
Timeline of events from time of presentation to last treatment cycle.

A timeline of events from this case can be referenced in **Figure 4**.

## Discussion

The pathogenesis of DDCS, specifically the immune microenvironment of these tumors, remains uncharacterized. Preclinical and clinical studies suggest that chondrosarcomas behave like inflammatory tumors with dense tumor-infiltrating lymphocytes and high expression of checkpoint inhibitor molecules such as PD-L1 (12–15). In an immunohistochemical analysis of 49 dedifferentiated chondrosarcoma tumor samples, Iseulys et al. identified PD-L1 positivity in up to 42% of patients (16). Similarly, among 22 dedifferentiated chondrosarcoma whole-tissue samples analyzed by Kostine et al., PD-L1 expression was seen in 52% of samples and was associated with high T-cell infiltration (17). Moreover, areas of high PD-L1 expression and high lymphocyte density colocalized to the dedifferentiated parts of the tumor, suggesting that immunotherapy could target the component of these tumors that are usually chemotherapy resistant. Though not well-established in chondrosarcomas, PD-L1 expression has also been correlated with survival in other sarcoma types, suggesting a possible survival benefit with immunotherapy in these patients (14, 18).

Due to the rarity of DDCS, clinical efficacy of immunotherapy in these patients is primarily inferred from data obtained in other sarcomas or described in a handful of case reports and case series. A small retrospective cohort study of metastatic sarcoma patients treated with the PD-1 inhibitor nivolumab, of whom two had chondrosarcoma, demonstrated partial response in one patient after six cycles of nivolumab and stable disease in the second patient after four cycles of nivolumab (19). Similarly, Wagner et al. described the case of a 67-year-old man with metastatic conventional chondrosarcoma with near-complete response following four cycles of nivolumab (20). The SARC028 trial, a multicenter phase II trial investigating the activity of pembrolizumab in patients with advanced soft-tissue and bone sarcoma, reported a partial objective response in only one of five patients with dedifferentiated chondrosarcoma (12). Unlike our patient with PD-L1-positive disease and robust response to pembrolizumab, PD-L1 expression was not present for all responders in these clinical studies.

Our patient had PD-L1 positivity in both tumor cells and infiltrating immune cells, suggesting that much remains to be understood about the tumor microenvironment of chondrosarcomas and if PD-L1 can be used as a biomarker or predictor of response to immunotherapy. Clinically, very few patients with sarcoma and specifically with chondrosarcomas respond to immunotherapy. Here, we describe a single case of a complete response to immunotherapy in a patient with dedifferentiated chondrosarcoma. Given this, we are limited in making broad statements regarding treatment of chondrosarcomas. Yet, in the literature, high levels of PD-L1 have been noted in dedifferentiated chondrosarcoma; others have reported treatment responses to immunotherapy, and response to other treatments such as chemotherapy or tyrosine kinase inhibitors is exceptionally low, making the consideration of immunotherapy in these patients reasonable.

## Patient perspective

When I received my diagnosis, my future seemed bleak. With the help of my oncologist, the decision was made to try pembrolizumab. I am still here 2 years later! I had minimal side effects. I have my own business and continue to work and thrive. And for this, I am so appreciative!

## Conclusion

DDCS are aggressive malignancies with devastating survival outcomes, especially in patients with metastatic disease. Tissue analyses from these patients suggest a molecular basis for the utility of checkpoint inhibition for systemic treatment. The rapid and durable response seen with PD-L1 inhibition in our patient indicates that immunotherapy may indeed be a successful treatment option in a subset of dedifferentiated chondrosarcoma patients.

## Data availability statement

The original contributions presented in the study are included in the article/supplementary material. Further inquiries can be directed to the corresponding author.

## Ethics statement

Ethical review and approval was not required for the study on human participants in accordance with the local legislation and institutional requirements. The patients/participants provided their written informed consent to participate in this study. Written informed consent was obtained from the individual(s) for the publication of any potentially identifiable images or data included in this article.

## Author contributions

All authors listed have made a substantial, direct, and intellectual contribution to the work, and approved it for publication.

## Funding

Janai Carr-Ascher is supported in part by the UC Davis Paul Calabresi Career Development Award for Clinical Oncology as funded by the National Cancer Institute/National Institutes of Health through grant #5K12- CA138464 as well as the UC Davis Cancer Center Support Grant P30CA093373.

## Conflict of interest

The authors declare that the research was conducted in the absence of any commercial or financial relationships that could be construed as a potential conflict of interest.

## Publisher’s note

All claims expressed in this article are solely those of the authors and do not necessarily represent those of their affiliated organizations, or those of the publisher, the editors and the reviewers. Any product that may be evaluated in this article, or claim that may be made by its manufacturer, is not guaranteed or endorsed by the publisher.
